# Specific circulating microRNAs during hepatitis E infection can serve as indicator for chronic hepatitis E

**DOI:** 10.1038/s41598-020-62159-9

**Published:** 2020-03-24

**Authors:** Dominik Harms, Mira Choi, Kristina Allers, Bo Wang, Heiko Pietsch, C.-Patrick Papp, Lina Hanisch, Jens Kurreck, Jörg Hofmann, C.-Thomas Bock

**Affiliations:** 1 Division of Viral Gastroenteritis and Hepatitis Pathogens and Enteroviruses, Department of Infectious Diseases, Robert Koch Institute, Berlin, Germany; 2 Medical Department, Division of Nephrology and Internal Intensive Care Medicine, Charité Universitätsmedizin Berlin, Berlin, Germany; 3 Medical Department, Division of Gastroenterology, Infectiology and Rheumatology (including Nutritional Medicine), Charité Universitätsmedizin Berlin, Berlin, Germany; 4 Department of Cardiology, Campus Rudolf Virchow, Charité Universitätsmedizin Berlin, Berlin, Germany; 5 DZHK (German Centre for Cardiovascular Research), Berlin, Germany; 6 Institute of Biotechnology, Technical University of Berlin, Berlin, Germany; 7 Institute of Medical Virology, Charité Universitätsmedizin Berlin, Berlin, Germany; 8 Labor Berlin, Charité-Vivantes GmbH, Berlin, Germany; 9 Institute of Tropical Medicine, University of Tübingen, Tübingen, Germany

**Keywords:** Microbiology, Virology, Viral host response

## Abstract

Hepatitis E virus (HEV) genotypes 3 and 4 (HEV-3, HEV-4) infections are an emerging public health issue in industrialized countries. HEV-3 and −4 are usually self-limiting but can progress to chronic hepatitis E in immunocompromised individuals. The molecular mechanisms involved in persistent infections are poorly understood. Micro RNAs (miRNAs) can regulate viral pathogenesis and can serve as novel disease biomarkers. We aimed to explore the modulation of serum miRNAs in patients with acute (AHE) and chronic (CHE) hepatitis E. Both AHE- and CHE-patients exhibited high viral loads (median 3.23E + 05 IU/mL and 2.11E + 06 IU/mL, respectively) with HEV-3c being the predominant HEV-genotype. Expression analysis of liver-specific serum miRNAs was performed using real-time PCR. miR-99a-5p, miR-122-5p, and miR-125b-5p were upregulated in AHE (4.70–5.28 fold) and CHE patients (2.28–6.34 fold), compared to HEV-negative controls. Notably, miR-192-5p was increased 2.57 fold while miR-125b-5p was decreased 0.35 fold in CHE but not in AHE patients. Furthermore, decreased miR-122-5p expression significantly correlates with reduced liver transaminases in CHE patients. To our knowledge, this marks the first investigation concerning the regulation of circulating liver-specific miRNAs in acute and chronic HEV infections. We found that miR-125b-5p, miR-192-5p, and miR-99a-5p may prove useful in the diagnosis of chronic hepatitis E.

## Introduction

Hepatitis E virus (HEV) is attributed to an estimated 20 million cases of acute viral hepatitis worldwide with 44,000 deaths each year, mainly occurring during large waterborne outbreaks of genotypes 1 and 2 (HEV-1, HEV-2)^1^. The fecal-orally transmitted HEV-1 and -2 are highly endemic in resource limited regions, such as Africa, India and Asia. Meanwhile, the number of reported autochthonous infections with HEV genotypes 3 and 4 (HEV-3, HEV-4) has risen exponentially over the last decade and hepatitis E is now recognized as an emerging disease in developed countries and a significant health burden^1–4^.

Infections in immunocompetent patients usually remain asymptomatic or present as a mild and self-limiting viral hepatitis without lasting damage. Serious complications such as fulminant hepatic failure can occur in patients with pre-existing liver disease while increased fetal and maternal mortality rates are reported in infected pregnant women^5,6^. Persistent infections can manifest in immunocompromised individuals including solid organ and hematopoietic stem cell transplant patients, leading to adverse clinical outcomes^7–9^. Viral RNA detectable in patient blood for a time period exceeding three months is considered indicative of chronic hepatitis E (CHE)^10^. The current off-label treatment option for CHE include the nucleoside analogue ribavirin but is often accompanied by clinical side effects^11^. In addition, evidence suggests that ribavirin may promote selection of viral quasi-species associated with treatment failure and viral recurrence^12–14^. The earliest detection of a persistent HEV infection is paramount to the advancement of successful antiviral treatment. In light of this, novel biomarkers for this emerging infectious disease need to be discovered and characterized.

Micro RNAs (miRNAs) are evolutionarily conserved ~21–25 nucleotide long single-stranded non-coding RNA molecules^15^. miRNAs are posttranscriptional regulators of gene expression through complementary binding to the 5′ or 3′UTR of target mRNA molecules^16^. More than 60% of human genes are predicted to be targets for endogenous miRNAs while a number of miRNAs are capable of targeting several mRNAs^17^. Consequently, miRNAs are involved in the regulation of numerous biological processes ranging from cell proliferation and apoptosis, tissue development and differentiation and immune responses^18^.

Several miRNA species have been found to be dysregulated in various diseases. The fact that tissue-specific miRNAs may enter the blood stream has opened up the possibility of using circulating miRNAs as non-invasive predictors of disease progression and treatment outcome^19,20^. Previous studies have investigated the modulation of miRNA levels in the setting of liver diseases such as drug- and alcohol-induced liver injury, as well as hepatocellular carcinoma^21–23^. However, miRNA expression is also affected by viral hepatitis like hepatitis B and hepatitis C. A number of miRNAs including miR-99a, miR-122, and miR-125b were found to be upregulated in patients with hepatitis B virus (HBV) infection and expression levels allowed differentiation between HBV, hepatitis C virus (HCV) and HBV-related hepatocellular carcinoma^24–27^. Additionally, circulating levels of miR-122, miR-125b and miR-192 were increased in HBeAg-positive patients^28^. Increased serum miR-122 levels are predictive for enhanced inflammation in chronic HCV patients^29,30^.

Only little is known concerning miRNA regulation during acute and chronic HEV infection. In acute hepatitis E miR-122, -221 and -222 have been implicated to regulate HEV *in vitro*, and several miRNAs expressed in peripheral blood mononuclear cells including miR-431, -654, -1468, and -4435 were able to differentiate acute hepatitis E in pregnant and non-pregnant^31–33^. A recent study has shown that HEV-encoded miRNAs may dysregulate host cell pathways during infection^34^. However, data of miRNA regulation in chronic hepatitis E are still missing. Therefore, we aimed to explore and compare the miRNA signatures in serum of acute and chronic HEV infected patients to elucidate differences in miRNA regulation which potentially may lead to chronic hepatitis E.

## Materials and Methods

### Patient samples

In total, 38 patient serum samples were included. Six samples were collected from patients presenting with acute hepatitis E infection (AHE) and viremia (AHEv), four from AHE patients without viremia (AHEnv). Twelve samples were from renal transplant patients suffering from chronic hepatitis E infection (CHE) with viremia (CHEv) and six from CHE patients without detectable HEV viral load (CHEnv) after ribavirin (RBV) treatment. Finally, ten samples from HEV sero- and RNA-negative renal transplant patients served as a control group (control). Inclusion criteria for AHE patients were detectable anti-HEV IgM antibodies and HEV RNA in blood (AHEv) or undetectable RNA (AHEnv), respectively. CHEv patients were tested positive for anti-HEV IgG and IgM and detectable HEV RNA in blood for more than three months. CHEv patients were tested retrospectively for HEV RNA up to two years prior to first detection by RT-PCR using stored serum samples. CHEnv patients had detectable anti-HEV IgG and IgM titers but were negative for HEV RNA in blood. Where applicable, patients were additionally tested for serum alanine (ALT) and aspartate transaminase (AST) levels.

### Molecular characterization of HEV

Viral RNA was extracted from 140 µl of serum using QIACube and the QIAamp Viral RNA Mini kit (Qiagen, Hilden, Germany) according to the manufacturer´s instructions. Viral loads were determined by HEV-specific real-time quantitative RT-PCR and HEV geno- and subtypes were determined by nested RT-PCR followed by Sanger sequencing and phylogenetic analysis using partial ORF1 and ORF2 sequences as previously described^35,36^.

### Profiling of circulating miRNAs

In order to evaluate miRNA expression levels and profiles of AHE, CHE groups, and HEV-negative control group, analysis of 180 miRNA species was performed using miRCURY LNA Universal cDNA Synthesis Kit II (Exiqon, Vedbaek, Denmark) for reverse transcription, ExiLENT SYBR Green master mix (Exiqon, Vedbaek, Denmark) for quantitative PCR (qPCR) amplification and Serum/Plasma Focus qPCR Panels V4.RO (Exiqon, Vedbaek, Denmark) according to the manufacturer´s instructions. As internal amplification controls synthetic miRNAs UniSp2, 4 and 5 were added as spike-ins before extraction and UniSp6 before cDNA synthesis (Exiqon, Vedbaek, Denmark). Data analysis was performed with GenEx qPCR analysis software (Exiqon, Vedbaek, Denmark). Raw cycle threshold (Ct) values were inter-plate calibrated using UniSp3. To identify endogenous reference miRNAs and regulated miRNAs for expression analyses during HEV infection, global mean normalization was performed. In brief, fold changes (FC) of miRNA expression in AHE and CHE patients compared to controls were calculated using the 2^−ΔΔCt^ method. Four regulated miRNAs were selected for further investigation based on previous studies^24–30^. The three most stably expressed miRNAs were selected as endogenous reference genes.

### Evaluation of miRNA profiles

Four regulated liver-specific miRNAs (miR-99a-5p, miR-122-5p, miR-125b-5p, miR-192-5p) and three potential reference miRNAs (let-7a-5p, let-7b-5p, miR-126-3p) were chosen for validation using miRNA-specific qPCR based on initial serum miRNA profiling. Expression levels of miRNAs of the panel were analyzed from 38 serum samples of 6 AHEv-, 4 AHEnv-, 12 CHEv-, 6 CHEnv-patients and 10 non-infected control patients.

Circulating miRNAs were extracted from 200 µl serum using the miRNeasy Serum/Plasma Advanced Kit (Qiagen, Hilden, Germany) following the manufacturer’s instructions. RNA was reverse transcribed using miRCURY LNA RT kit (Qiagen, Hilden, Germany) following the protocol for 10 µl reactions using 1 µl of RNA. Expression analysis via miRNA-specific qPCR was performed using 2x miRCURY SYBR Green Master Mix (Qiagen, Hilden, Germany) and miRCURY LNA miRNA Custom 96 well PCR Panels (Qiagen, Hilden, Germany) on a Roche LightCycler 480 II instrument (Roche Diagnostics GmbH, Mannheim, Germany) according to manufacturer instructions. Non-template controls (NTC) were used to assess background fluorescence and reasonable cut-off values for expression analysis.

### Statistical analysis

Raw Ct values were calculated using absolute quantification and uploaded to the Qiagen GeneGlobe Data Analysis online tool (https://www.qiagen.com/us/shop/analytics-software/biological-data-tools/geneglobe-data-analysis-center/). A cut-off of Ct = 36 was chosen to ensure inclusion of only robustly amplified samples based on non-template control values.

Three potential endogenous reference miRNAs (let-7a-5p, let-7b-5p, miR-126-3p) were evaluated based on the premise that the ratio of expression of two true reference miRNAs is identical across all patient groups^37^. ΔCt values were calculated using the average of the two most stable reference miRNAs and fold changes of each regulated miRNA species determined via the 2^−ΔΔCt^ method. Statistical analyses were performed with standard deviations of ΔΔCt values using unpaired, two-tailed Student’s t-test with p-values below 0.05 considered as statistically significant.

Scatter plots, ROC, AUC and correlation analysis were designed using GraphPad Prism v8.1.2 (GraphPad Software, Inc., San Diego, USA). Receiver operating characteristic (ROC) was applied to test predictive performance of liver-specific miRNAs. ROC area under curve (AUC) was calculated by DeLong method. Optimal cut-off values were determined by Youden’s index (Supplementary Tables S2 and S3). Pearson correlation coefficient was calculated for correlation analysis of miR-122-5p and ALT/AST levels. Two-tailed p-values were calculated. P-values below 0.05 were considered to indicate statistical significance.

### Ethical approval

The local ethics committee of the Charité Universitätsmedizin Berlin approved the study (approval number No. EA1/367/16) and written informed consent was obtained. Patient samples were de-identified for this study. All experiments were performed in accordance with relevant guidelines and regulations.

## Results

### Patient characteristics and determination of HEV infection

All 38 patient samples included were analyzed for virus load and (sub)genotype using HEV-specific real-time qPCR and nested RT-PCR followed by Sanger sequencing and phylogenetic analyses. AHEv and CHEv serum samples were found to be viremic with a median viral load of 3.23E + 05 IU/ml serum (1.44E + 04 to 8.15E + 06 IU/ml) and 2.11E + 06 IU/ml (1.29E + 04 to 7.27E + 06 IU/ml), respectively. Phylogenetic analyses showed that all HEV-positive samples were HEV genotype 3. 5/6 AHEv and 9/12 CHEv clustered within HEV subtype 3c while one AHEv and one CHEv sample belonged to subtype 3e. The remaining two CHEv samples clustered within subtype 3b and 3f, respectively. Patient details with clinical and virological characteristics are summarized in Table 1.

**Table 1:** Clinical and virological characteristics of HEV-positive patients and HEV-negative controls.

Sample ID	Gender (F/M)	% F/M	Age (y)	Age (y) ∅	Trans-plant status	HEV status	1^st^ HEV RNA detection (d)	1^st^ HEV RNA detection (d) ∅	ALT/AST [IU/l]	anti-HEV IgG/IgM	Viral load [IU/ml]	Viral Load [IU/mL] ∅	HEV genotype
DE/17–0463	F	50/50	73	58	none	AHEv	0	0	N/A	+/+	8.15E + 06	3.23E + 05	3c
DE/18–0223	F		78				0		52/58	+/+	3.55E + 05		3c
DE/18–0224	M		54				0		1417/383	−/+	2.91E + 05		3c
DE/18–0225	F		37				0		54/44	+/+	4.57E + 05		3e
DE/18–0226	M		46				0		155/131	+/+	2.36E + 05		3c
DE/18–0227	M		62				0		65/36	+/+	1.44E + 04		3c
DE/18–0218	M	50/50	65	44.5	none	AHEnv	—	—	392/390	+/+	—	—	N/A
DE/18–0219	F		33				—		121/41	+/+	—		N/A
DE/18–0220	M		56				—		N/A	+/+	—		N/A
DE/18–0222	F		32				—		926/1522	+/+	—		N/A
DE/16–0010	F	25/75	61	55	renal	CHEv	830	732	29/29	+/+	9.41E + 05	2.11E + 06	3c
DE/16–0013	M		46				810		285/121	+/+	3.22E + 06		3f
DE/16–0014	M		54				673		85/56	+/+	5.96E + 05		3e
DE/16–0015	F		34				1314		21/19	+/+	2.91E + 06		3c
DE/16–0017	M		25				401		116/36	+/+	6.63E + 05		3c
DE/16–0018	M		56				561		212/96	+/+	1.79E + 06		3c
DE/16–0019	M		52				91		159/88	+/+	2.16E + 06		3c
DE/16–0020	M		54				848		62/44	+/+	4.20E + 06		3c
DE/16–0022	M		57				926		62/52	+/+	2.07E + 06		3b
DE/16–0023	F		60				282		32/37	+/+	4.70E + 06		3c
DE/16–0024	M		76				791		57/62	+/+	1.29E + 04		3c
DE/17–0464	M		56				607		189/96	+/+	7.27E + 06		3c
DE/17–0465	M	20/80	57	57.5	renal	CHEnv	691	866	44/31	+/+	—	—	N/A
DE/18–0228	M		47				1010		16/27	+/+	—		N/A
DE/18–0229	M		58				1015		34/27	+/+	—		N/A
DE/18–0230	F		61				539		11/17	+/+	—		N/A
DE/18–0232	M		56				722		21/23	+/+	—		N/A
DE/18–0233	M		76				1050		18/29	+/+	—		N/A
DE/18–0234	F	50/50	36	58	renal	HEV-negative	—	—	12/24	−/−	—	—	N/A
DE/18–0235	M		68				—		9/23	−/−	—		N/A
DE/18–0236	F		47				—		17/21	−/−	—		N/A
DE/18–0237	F		57				—		16/17	−/−	—		N/A
DE/18–0238	M		67				—		22/22	−/−	—		N/A
DE/18–0239	M		50				—		21/18	−/−	—		N/A
DE/18–0240	M		62				—		28/28	−/−	—		N/A
DE/18–0241	F		58				—		16/25	−/−	—		N/A
DE/18–0242	M		59				—		25/23	−/−	—		N/A
DE/18–0243	F		58				—		109/42	−/−	—		N/A

N/A = not applicable; ∅ = median; y = years, d = days; ALT/AST = transaminases, AHEv = viremic acute; AHEnv = non-viremic acute; CHEv = viremic chronic; CHEnv = non-viremic chronic.

9/12 CHEv patients had increased transaminase levels (defined as >35 IU/ml), whereas only 1/6 CHEnv and 1/10 HEV-negative control patients had moderately increased ALT/AST levels. Transaminase levels determined in 5/6 AHEv patients showed increased ALT/AST values. Similarly, all AHEnv patients for which transaminase levels were available (3/4) exhibited increased ALT/AST levels (Table 1).

### Identification of regulated miRNAs

A preliminary miRNA expression profiling of 180 common circulating miRNAs using Serum/Plasma Focus qPCR Panels V4.RO was performed with miRNAs isolated from serum of a AHEv, AHEnv, CHEv, CHEnv and non-HEV renal transplant control patient, respectively (Supplementary Table S1). Regulation of expression levels of miRNAs in HEV-positive samples compared to the control group was observed for liver-specific miRNAs. From these, miR-99a-5p, miR-122-5p, miR-125b-5p and miR-192-5p were chosen for validation based on their involvement in HBV and HCV described in previous studies^24–30^. Three none or only minor regulated miRNAs (let-7a-5p, let-7b-5p and miR-126-3p) among all tested miRNAs in AHEv, AHEnv, CHEv and CHEnv when compared to non-HEV controls were selected to validate their use as endogenous reference miRNAs in HEV infection.

### let-7a-5p and let-7b-5p serve as serum reference miRNAs in HEV infection

To evaluate the three candidate reference genes identified in the preliminary miRNA profiling, their expression ratios were analyzed. Normalization was carried out with each of the three potential reference miRNAs. Fold changes (FCs) compared to HEV-negative controls were calculated for each data set. When let-7a-5p was used for normalization, let-7b-5p expression exhibited stable expression among AHEnv (FC = 0.99), CHEv (FC = 0.93), CHEnv (FC = 0.97) and AHEv (FC = 0.77) patients. Conversely, normalization with let-7b-5p resulted in stable expression of let-7a-5p among AHEnv (FC = 1.01), CHEv (FC = 1.08), CHEnv (FC = 1.04) and AHEv (FC = 1.30) patients as well (Table 2). Let-7a-5p and let-7b-5p thereby possessed the most stable expression ratios among the three tested miRNAs in AHEnv, CHEv and CHEnv serum samples and thereby proved to be the most optimal candidates serving as endogenous reference genes in HEV infection.

**Table 2:** Fold changes of potential endogenous serum reference miRNAs let-7a-5p and let-7b-5p in HEV patients compared to non-infected control group.

	AHEv		AHEnv		CHEv		CHEnv	
**Fold changes after normalization with let-7a-5p**								
miRNA	FC	p-value	FC	p-value	FC	p-value	FC	p-value
let-7b-5p	0.77	0.138	0.99	0.816	0.93	0.705	0.97	0.656
miR-126-3p	2.06	<0.001	2.00	<0.001	1.61	0.043	2.00	<0.001
	**AHEv**		**AHEnv**		**CHEv**		**CHEnv**	
**Fold changes after normalization with let-7b-5p**								
miRNA	FC	p-value	FC	p-value	FC	p-value	FC	p-value
let-7a-5p	1.30	0.130	1.01	0.922	1.08	0.524	1.04	0.961
miR-126-3p	2.67	<0.001	2.02	0.003	1.73	0.016	2.07	<0.001
	**AHEv**		**AHEnv**		**CHEv**		**CHEnv**	
**Fold changes after normalization with let-7a-5p and let-7b-5p in combination**								
miRNA	FC	p-value	FC	p-value	FC	p-value	FC	p-value
let-7a-5p	1.14	0.124	1.01	0.990	1.04	0.562	1.02	0.878
let-7b-5p	0.88	0.127	0.99	0.875	0.96	0.656	0.98	0.722
miR-126-3p	2.34	<0.001	2.01	<0.001	1.67	0.019	2.03	<0.001

FC = fold change; AHEv = viremic acute; AHEnv = non-viremic acute; CHEv = viremic chronic; CHEnv = non-viremic chronic.

### Liver-specific miRNAs are differentially regulated in acute and chronic HEV infections

Initial analyses revealed that miR-99a-5p, miR-122-5p, miR-125b-5p and miR-192-5p were most promising candidates to differentiate between acute and chronic hepatitis E by miRNA signatures. To further analyze these candidate miRNAs, detailed expression analyses were conducted on serum miRNAs from AHEv, AHEnv, CHEv, CHEnv and non-HEV controls.

**Figure 1:**
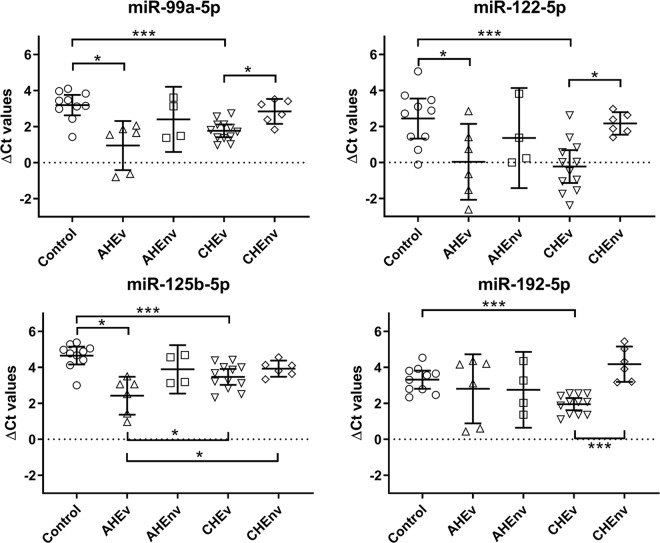
Scatter plot of ΔCt values of liver-specific miRNA in serum of AHE and CHE patients and control group depicted in scatter plots. Each dot represents one patient sample. Bars represent 95% confidence intervals of the mean. Statistically significant differences between patient groups depicted with asterisks (* < 0.05; ** < 0.01; *** < 0.001) and determined using standard deviation of ΔΔCt values.

Expression of candidate miRNAs was normalized using the mean Ct of let-7a and let-7b. Plotted mean ΔCt values with 95% confidence intervals of the 4 target miRNAs are depicted in Fig. 1.

Expression of liver-specific serum miRNAs in AHE and CHE was first compared to the non-infected control group (Table 3). MiR-99a-5p showed strongest regulation in serum of AHEv with a fold change (FC) of 4.74 (p-value = 0.014) compared to the control group followed by CHEv with FC of 2.69 (p-value < 0.001). Similarly, miR-125b-5p showed highest expression in serum of AHEv and CHEv with FCs of 4.70 (p-value = 0.004) and 2.28 (p-value = 0.006), respectively. Serum expression of miR-122-5p was increased 6.34-fold (p-value = 0.008) in CHEv and 5.28-fold (p-value = 0.038) in AHEv compared to the control. Additionally, CHEv patients exhibited a 2.57-fold increase (p-value < 0.001) of serum miR-192-5p. Although no statistically significant regulation of the mean expression of investigated serum miRNAs was observed in AHEnv and CHEv when compared to the control group, a subset of AHEnv patients retained increased levels of miR-122-5p (p-value = 0.219).

**Table 3:** Fold changes of liver-specific serum miRNAs in HEV patients compared to non-infected control group.

miRNA	AHEv		AHEnv		CHEv		CHEnv	
	FC	p-value	FC	p-value	FC	p-value	FC	p-value
miR-99a-5p	4.74	0.014	1.73	0.139	2.69	<0.001	1.27	0.600
miR-122-5p	5.28	0.038	2.12	0.219	6.34	0.008	1.21	0.648
miR-125b-5p	4.70	0.004	1.70	0.136	2.28	0.006	1.66	0.118
miR-192-5p	1.42	0.127	1.48	0.130	2.57	<0.001	0.55	0.081

FC = fold change; AHEv = viremic acute; AHEnv = non-viremic acute; CHEv = viremic chronic; CHEnv = non-viremic chronic.

Next, expression of the candidate miRNAs in chronic HEV group was compared to acute HEV group (Table 4). Lower expression levels of miR-125b-5p were observed when comparing CHEv and CHEnv to AHEv with FCs of 0.48 (p-value = 0.024) and 0.35 (p-value = 0.043), respectively. In addition, a subset of CHEnv patients had lower serum levels of miR-99a-5p (FC = 0.27, p-value = 0.067), miR-122-5p (FC = 0.23, p-value = 0.096) and miR-192-5p (FC = 0.39, p-value = 0.143), although mean expression in these patients was not significantly reduced. No significant miRNA regulation was observed in CHEv and CHEnv when compared to AHEnv, however, miR-192-5p was downregulated in a subset of CHEnv patients (FC = 0.37, p-value = 0.071) while a subset of CHEv patients demonstrated increased miR-122-5p levels (FC = 2.99, p-value = 0.160).

**Table 4:** Fold changes of liver-specific serum miRNAs in chronic HEV patients compared to acute HEV patients.

miRNA	vs. AHEv				vs. AHEnv			
	CHEv		CHEnv		CHEv		CHEnv	
	FC	p-value	FC	p-value	FC	p-value	FC	p-value
miR-99a-5p	0.57	0.049	0.27	0.067	1.55	0.290	0.74	0.295
miR-122-5p	1.20	0.806	0.23	0.096	2.99	0.160	0.57	0.091
miR-125b-5p	0.48	0.024	0.35	0.043	1.34	0.396	0.97	0.680
miR-192-5p	1.81	0.962	0.39	0.143	1.74	0.259	0.37	0.071

FC = fold change; AHEv = viremic acute; AHEnv = non-viremic acute; CHEv = viremic chronic; CHEnv = non-viremic chronic.

Finally, differences in serum miRNA levels between viremic and non-viremic HEV patients were investigated (Table 5). While no statistically significant regulation in mean serum expression could be identified in AHEnv compared to AHEv patients, a subset of AHEnv patients demonstrated downregulated miR-99a-5p (FC = 0.37, p-value = 0.198), miR-122-5p (FC = 0.40, p-value = 0.278) and miR-125-p (FC = 0.36, p-value = 0.120) levels. However, compared to CHEv patients, CHEnv patients exhibited significantly decreased expression of serum miR-99a-5p, miR-122-5p and miR-192-5p with FCs of 0.47 (p-value = 0.007), 0.19 (p-value = 0.029) and 0.21 (p-value < 0.001), respectively.

**Table 5:** Fold changes of liver-specific serum miRNAs in viremic compared to non-viremic HEV patients.

miRNA	vs. AHEv AHEnv		vs. CHEv CHEnv	
	FC	p-value	FC	p-value
miR-99a-5p	0.37	0.198	0.47	0.007
miR-122-5p	0.40	0.278	0.19	0.029
miR-125b-5p	0.36	0.120	0.73	0.148
miR-192-5p	1.04	0.652	0.21	<0.001

FC = fold change; AHEv = viremic acute; AHEnv = non-viremic acute; CHEv = viremic chronic; CHEnv = non-viremic chronic.

### Statistical analyses on predictive performances of investigated liver-specific miRNAs in acute and chronic hepatitis E patients

The predictive value to distinguish between CHEv or AHEv and non-infected control patients of miR-99a-5p, miR-125b-5p and miR-192-5p was assessed. ROC AUC values were calculated from the curves to compare the performance of each miRNA in a singular test. In serum of AHEv patients, only miR-99a-5p (ROC AUC = 0.933; p-value = 0.005) and miR-125b-5p (ROC AUC = 0.950; p-value = 0.003) proved significant predictive performance. However, in CHEv patients miR-99a-5p (ROC AUC = 0.917; p-value = 0.001), miR-125b-5p (ROC AUC = 0.900; p-value = 0.002), as well as miR-192-5p (ROC AUC = 0.942; p-value < 0.001) demonstrated to be predictive for chronic HEV when compared to HEV-negative control patients (Table 6). These data are in accordance with miRNA regulation in AHEv and CHEv patients when compared to control patients (Table 3). ROC curves of the investigated miRNAs in AHEv and CHEv patients are presented in Supplementary Fig. S4.

**Table 6:** Predictive values of miR-99a-5p, miR-125b-5p and miR-192-5p in AHEv and CHEv patients compared to non-infected control group as calculated by ROC analysis.

HEV status	miRNA	ROC AUC	SEM	95% CI [%]	p-value	Sensitivity	Specificity
CHEv	miR-99-5p	0.917	0.068	0.783–1.051	0.001	83.3%	80%
	miR-125-5p	0.900	0.076	0.751–1.049	0.002	83.3%	80%
	miR-192-5p	0.942	0.048	0.848–1.036	<0.001	75%	80%
AHEv	miR-99-5p	0.933	0.069	0.799–1.068	0.005	100%	90%
	miR-125-5p	0.950	0.055	0.842–1.058	0.003	100%	90%
	miR-192-5p	0.517	0.179	0.167–0.867	0.914	50%	0%

AHEv = viremic acute; CHEv = viremic chronic; ROC AUC = receiver operating characteristic area under curve; SEM = standard error of mean; CI = confidence interval; Sensitivity and specificity are given for fixed values of 90% specificity and 90% sensitivity, respectively.

Correlation analysis of miR-122-5p expression in CHEv, CHEnv and non-infected control patients demonstrated a significant correlation between reduced miR-122-5p expression and decreased levels of ALT (Pearson r = −0.552; p-value = 0.002), as well as AST (Pearson r = −0.580; p-value = 0.001) (Table 7) in serum of these groups. Graphs of correlation analysis can be found in Supplementary Fig. S5.

**Table 7:** Correlation analysis of serum miR-122-5p expression and ALT/AST levels in CHE and non-infected control patients.

∆Ct miR-122-5p vs. ALT/AST			
Parameter	Pearson r	95% CI [%]	p-value
ALT	−0.552	(−0.767)–(−0.220)	0.002
AST	−0.580	(−0.784)–(−0.260)	0.001

ALT = alanine transaminase; AST = aspartate transaminase; CHE = chronic hepatitis E; CI = confidence interval.

## Discussion

HEV infection is a major cause of acute hepatitis globally showing various clinical outcomes from asymptomatic courses to severe hepatitis. Chronic hepatitis E is recognized as a growing health issue in immunocompromised patients.

It is now recognized that renal transplant patients are at risk of developing chronic hepatitis E; however, the full spectrum of adverse effects is still unclear. A retrospective study in German renal transplant recipients determined a 1.1% (16 out of 1469) prevalence of chronic HEV and 16.3% prevalence among those patients with elevated liver enzymes (16 out of 98)^8^. As the time point of seroconversion of HEV is variable, molecular methods should be used to diagnose HEV infection^8^. However, neither serology nor nucleic acid detection have yet conclusively identified factors involved in development of chronicity or disease courses. In light of this, we aimed to determine regulated miRNAs in serum of patients with acute and chronic manifestations of HEV infection to elucidate differences and discover potential indicators of disease progression.

MiRNAs are known to modulate viral pathogenesis and are therefore involved in determining the course of infection. Our study marks the first investigation of the regulation of circulating miRNAs in patients with acute versus chronic hepatitis E, including viremic and non-viremic HEV infections.

All isolated HEV RNA from patient serum included in the study belonged to genotype 3, with subtype 3c being predominant. A previous study found no association between disease severity and HEV-3 subtypes and our findings further ensured exclusion of differences in serum miRNA expression occurring from HEV genotypes^38^. Furthermore, to our knowledge there are no documented pharmacological effects of immunosuppressive drugs on the expression of the hepatic miRNAs investigated in this work. Therefore, comparisons between miRNA profiles in acute and chronic hepatitis E patients are deemed valid.

Initial profiling of 180 serum miRNAs showed potential regulation of the liver-specific miRNAs miR-99a-5p, miR-122-5p, miR-125b-5p and miR-192-5p (suppl. Table S1). As only one patient sample per group was initially tested, discrepancies compared to the validation cohort can be explained by individual miRNA expression levels as it is evident from Fig. 1. However, validation of these candidates confirmed significant differences in miRNA expression profiles in serum of HEV infected patients (Tables 3–5). Although miRNA spike-ins were included during profiling, these do not reflect the mean circulating miRNA content or quality^39,40^. It has previously been suggested to screen for potential endogenous reference miRNAs based on their expression stability and then further validate these^41^. Two of the three potential reference miRNAs identified during profiling, let-7a-5p and let-7b-5p, showed comparable expression levels across all patient groups in the validation phase and thus were used for normalization of expression data (Table 2). Notably, let-7a-5p has also been identified as a reference miRNA in serum of liver carcinoma patients suggesting that its expression levels remain unaltered during liver disease^42^.

Changes in circulating miRNA signatures have been studied for different clinical manifestations in both HBV and HCV infected patients^24–27,29,30^. Our results revealed differences in serum miRNA regulation between HEV infected and non-HEV patients, as well as acute and chronic, patients (Tables 3–5). Particularly, the combination of miR-99a-5p, -122-5p and -125b-5p for AHEv and miR-99a-5p, -122-5p, -125b-5p and -192-5p for CHEv may be used to differentiate acute from chronic infections when comparing to non-HEV controls (Table 3).

When comparing AHE and CHE (Table 4), miRNA expression levels of miR-125-5p showed a significant decrease in CHEv and CHEnv if compared to AHEv. Additionally, miR-99a-5p was decreased in CHEv (FC = 0.57, p-value = 0.049) as well as in a subset of CHEnv (FC = 0.27, p-value = 0.067). Therefore, miR-125b-5p and miR-99a-5p may be among those miRNAs whose expressions are altered during chronic infection. However, to specify whether transplant patients have lower levels of miR-99a-5p and miR-125b-5p as well as higher levels of miR-192-5p than non-transplanted acute patients at onset of the HEV infection or if these changes occur after transition of acute to persistent infection requires further examination in a larger AHE and CHE cohort. Additionally, miRNA profiles during acute and chronic phases within the same patient should be investigated in more detail in further experiments. However, these experiments were not possible in this analysis due to the limited number of available samples.

In addition, miR-122-5p expression is increased in CHEv patients compared to HEV-negative renal transplant controls (FC = 6.34, p-value = 0.008), whereas CHEnv patients showed comparable expression levels to control patients (FC = 1.21, p-value = 0.648). Previous reports have shown that increased miR-122-5p serum levels could be associated with various liver injuries and are considered as potential biomarkers^43,44^. As 9/12 CHEv patients had increased transaminase levels at time of sample collection, compared to only 1/6 CHEnv and 0/10 non-HEV renal transplant control patients, a correlation between increased miR-122-5p expression and liver injury appears obvious. Indeed, correlation analysis on miR-122-5p expression and ALT/AST levels in these patient groups confirm a significant correlation of these clinical parameters. This finding merits further investigation in a larger patient cohort.

The role of the investigated miRNAs during HEV infection remains unclear, however hypotheses based on the reported functions of miR-99a-5p, -122-5p, -125b-5p and -192-5p can be made. Common described functional activity of these miRNAs is the maintenance of hepatic differentiation and function, as well as suppression of cell proliferation and apoptosis^45–50^. Additionally, involvement of miR-122-5p in viral replication is demonstrated by its stabilization of the HCV genome and subsequent enhancement of replication^51^. Notably, a recent *in vitro* study has shown a similar effect of miR-122-3p on HEV replication^32^. As 3p and 5p forms are processed from a common pre-miRNA, the 3p form may be retained in the liver during HEV infection to promote replication while the 5p form is sequestered into the serum. HEV may profit from increased hepatic cell maintenance and decreased apoptosis, allowing lasting intrahepatic replication as well as from a possible pro-viral role of miR-122. However, such potential mechanisms must be confirmed using liver tissue from hepatitis E patients or suitable *in vitro* models.

In conclusion, this study marks the initial report of miRNA signatures determined in serum from acute and chronic hepatitis E virus infected patients. It is to our knowledge the first description of let-7a-5p and let-7b-5p as reference miRNAs for use in HEV serum studies. The regulated miRNAs miR-99a-5p, miR-122-5p, miR-125b-5p and miR-192-5p are not only important in the liver setting but share functional properties such as suppression of cell proliferation as well as apoptosis and may possibly play pro-viral roles in HEV replication. In our observation, miR-192-5p expression with accompanied viral load in blood may differentiate between acute and chronic stages of HEV infection compared to non-HEV patients. In addition, miR-125-5p and miR-99a-5p may serve as additional indicators to differentiate between acute viremic and persistent HEV infection. The predictive value of these three miRNAs were analyzed using ROC curves and found to be significant with miR-192-5p expression in particular being able to differentiate acute (ROC AUC = 0.517; p-value = 0.914) and chronic (ROC AUC = 0.942; p-value < 0.001) phases of HEV infection (Table 6). We further observed a significant correlation of miR-122-5p and transaminase levels (ALT/AST) in CHE and HEV-negative renal transplant control patients. Further studies are needed to pinpoint the temporal modulation of miRNA expression during transition from acute to chronic hepatitis E. The differentiation between acute and chronic infection at the earliest time point is crucial for fast and effective patient management and it is undeniable that miRNAs, as a class of emerging and non-invasive biomarkers, will play a leading role in future diagnostics.

## Supplementary information

Dataset 1.

## Data Availability

All data generated or analysed during this study are included in this article and in the supplementary material.
