# Supplementary material for: Specific circulating microRNAs during hepatitis E infection can serve as indicator for chronic hepatitis E
Source: Sci Rep. 2020 Mar 24;10:5337. doi: 10.1038/s41598-020-62159-9 (PMC7093451; doi:10.1038/s41598-020-62159-9)

Supplementary Information

Specific circulating microRNAs during hepatitis E infection can serve as indicator for chronic hepatitis E

Dominik Harms[1], Mira Choi[2], Kristina Allers[3], Bo Wang[1], Heiko Pietsch[4], C.-Patrick Papp[1], Lina Hanisch[1], Jens Kurreck[5], Jörg Hofmann[6,7], C.-Thomas Bock[1,8,*]

[1]Division of Viral Gastroenteritis and Hepatitis Pathogens and Enteroviruses, Department of Infectious Diseases, Robert Koch Institute, Berlin, Germany

[2]Medical Department, Division of Nephrology and Internal Intensive Care Medicine, Charité Universitätsmedizin Berlin, Berlin, Germany

[3]Medical Department, Division of Gastroenterology, Infectiology and Rheumatology (including Nutritional Medicine), Charité Universitätsmedizin Berlin, Berlin, Germany

[4]Department of Cardiology, Campus Rudolf Virchow, Charité Universitätsmedizin Berlin, Berlin, Germany

[5]Institute of Biotechnology, Technical University of Berlin, Berlin, Germany

[6]Institute of Medical Virology, Charité Universitätsmedizin Berlin, Berlin, Germany

[7]Labor Berlin, Charité-Vivantes GmbH, Berlin, Germany

[8]Institute of Tropical Medicine, University of Tübingen, Tübingen, Germany

*Corresponding author: C.-Thomas Bock, BockC@rki.de

Supplementary Table S1 Fold changes of serum miRNAs in HEV patients compared to non-infected control group from initial profiling

	AHEv	AHEnv	CHEv	CHEnv		AHEv	AHEnv	CHEv	CHEnv
miRNA	FC	FC	FC	FC	miRNA	FC	FC	FC	FC
miR-652-3p	0.06	0.34	0.96	0.10	miR-125b-5p	1.99	0.12	0.46	0.38
miR-221-3p	1.90	0.16	0.59	0.05	miR-192-5p	0.90	0.19	0.18	0.09
let-7f-5p	1.19	0.04	0.69	0.61	miR-335-5p	2.27	0.79	0.97	2.03
miR-27b-3p	1.69	0.38	0.59	1.43	miR-320d	3.69	1.93	1.05	0.47
miR-374b-5p	2.47	5.61	2.26	0.67	miR-22-3p	7.69	0.45	0.07	0.04
miR-93-5p	1.14	1.09	0.41	0.02	miR-199a-3p	1.77	0.49	0.97	3.21
miR-484	2.13	0.54	1.03	0.05	miR-19b-3p	1.74	0.60	0.40	0.89
miR-106a-5p	1.32	1.60	0.45	1.34	miR-29a-3p	3.47	0.24	0.54	0.87
miR-486-5p	1.29	2.62	0.44	1.79	miR-30b-5p	0.75	0.82	0.49	0.74
miR-26a-5p	0.85	2.87	0.64	0.99	miR-409-3p	0.45	0.66	0.80	1.60
miR-143-3p	0.26	0.50	0.37	5.66	miR-505-3p	2.01	1.14	0.94	3.02
miR-30a-5p	2.85	4.05	0.87	1.00	miR-361-5p	1.09	0.24	1.00	1.16
miR-342-3p	0.41	0.85	1.32	1.33	miR-27a-3p	2.14	2.36	1.08	2.26
miR-185-5p	1.64	1.03	0.56	3.25	miR-32-5p	1.70	0.26	0.28	1.69
let-7e-5p	0.35	2.46	0.44	1.21	let-7d-5p	1.40	1.94	0.77	0.12
miR-139-5p	1.07	1.73	2.42	2.45	miR-106b-5p	0.02	1.28	0.05	0.71
miR-451a	2.16	1.02	0.73	2.30	miR-328-3p	0.77	0.42	0.51	1.86
miR-128-3p	1.19	0.26	0.32	1.05	miR-15a-5p	1.90	0.23	0.43	1.57
miR-125a-5p	1.38	1.65	2.37	1.74	miR-532-5p	0.26	0.55	0.55	0.98
miR-25-3p	1.72	2.62	0.64	1.95	miR-194-5p	0.39	0.49	0.14	0.03
miR-375	2.61	0.71	0.87	0.55	miR-574-3p	0.72	0.42	0.51	0.42
miR-33a-5p	1.02	0.37	1.12	1.78	miR-320c	2.20	0.65	0.67	0.43
miR-16-5p	1.71	0.69	0.47	1.39	miR-130a-3p	1.05	0.62	0.28	0.30
miR-152-3p	4.73	0.64	0.28	1.65	miR-28-5p	0.93	0.44	0.78	0.68
miR-197-3p	0.94	0.70	1.63	4.66	let-7c-5p	0.06	2.48	0.41	0.51
miR-146a-5p	0.87	0.40	0.49	1.43	miR-29b-3p	0.10	0.22	0.69	0.91
miR-424-5p	2.06	0.17	0.81	4.89	miR-1260a	0.05	16.55	0.13	0.98
miR-107	1.30	1.54	0.45	0.91	miR-100-5p	26.87	1.77	1.77	1.98
miR-148b-3p	2.47	0.18	0.63	2.20	miR-339-5p	0.64	0.29	0.67	1.10
miR-30d-5p	2.37	0.72	0.63	1.37	miR-378a-3p	3.03	0.08	0.58	1.43
let-7i-5p	1.35	1.85	0.67	0.07	miR-425-5p	2.04	1.18	0.70	2.72
miR-26b-5p	0.93	2.25	0.46	0.97	miR-454-3p	0.81	0.51	0.30	0.72
miR-320b	2.47	0.79	0.84	0.52	miR-874-3p	0.41	0.84	1.02	0.65
miR-590-5p	7.07	1.81	1.86	7.99	miR-885-5p	9.64	0.28	0.34	0.21
miR-191-5p	1.55	2.31	0.82	1.55	miR-16-2-3p	2.14	1.63	0.88	1.64

miR-99a-5p	3.00	0.28	0.29	0.18	miR-140-5p	2.07	0.93	0.68	1.23
miR-301a-3p	1.08	1.38	0.42	0.84	miR-629-5p	0.66	0.24	0.30	0.53
miR-122-5p	0.48	0.11	0.07	0.08	miR-423-3p	1.69	0.75	1.19	2.16
miR-423-5p	1.76	0.10	1.16	1.71	miR-376a-3p	0.35	0.98	0.86	1.55
miR-101-3p	2.48	1.47	0.67	3.58	miR-22-5p	0.16	0.33	0.40	0.26
miR-365a-3p	0.71	0.44	0.53	0.34	miR-93-3p	0.48	0.84	1.03	0.79
miR-23a-3p	1.71	0.46	0.01	1.99	miR-144-5p	3.78	3.50	1.02	2.34
miR-215-5p	0.54	0.51	0.14	0.19	miR-210-3p	4.92	0.28	0.34	0.22
miR-320a	2.25	0.53	0.73	0.49	miR-199a-5p	1.01	0.18	0.35	1.11
miR-338-3p	1.02	0.63	0.76	3.92	miR-363-3p	1.44	1.17	0.31	0.58
miR-103a-3p	1.27	0.46	0.47	0.76	miR-374a-5p	1.08	14.31	1.04	0.76
miR-331-3p	0.74	0.21	0.34	0.62	miR-151a-3p	3.20	0.89	0.84	0.53
let-7d-3p	1.62	0.18	0.34	1.46	miR-324-3p	6.49	3.20	2.63	2.10
miR-126-5p	0.05	2.49	1.36	1.21	miR-15b-5p	1.06	2.58	0.06	1.30
miR-19a-3p	1.52	1.22	0.42	0.80	miR-29c-3p	3.89	0.64	0.64	0.79
miR-148a-3p	7.58	0.33	0.68	3.89	miR-21-5p	4.63	0.99	0.84	2.68
miR-10b-5p	1.03	1.03	2.94	0.94					

FC = fold change; AHEv = viremic acute; AHEnv = non-viremic acute; CHEv = viremic chronic; CHEnv = non-viremic chronic

Supplementary Table S2 Optimal cut-off values for miR-99a-5p, miR-125b-5p and miR-192-5p in serum samples from AHEv patients when compared to non-infected control patients as determined by Youden's Index for ROC analysis

			AHEv				
miRNA	Cut-off	Sensitivity [%]	95% CI [%]	Specificity [%]	95% CI [%]	Likelihood Ratio	Youden's J
miR-99a-5p	< -0.710	16.67	0.42 - 64.12	100	69.15 - 100		0.167
	< 0.405	33.33	4.33 - 77.72	100	69.15 - 100		0.333
	< 1.490	33.33	4.33 - 77.72	90	55.50 - 99.75	3.333	0.233
	< 1.600	50	11.81 - 88.19	90	55.50 - 99.75	5.000	0.400
	< 1.755	66.67	22.28 - 95.67	90	55.50 - 99.75	6.667	0.567
	< 1.960	83.33	35.88 - 99.58	90	55.50 - 99.75	8.333	0.733
	< 2.250	100	54.07 - 100	90	55.50 - 99.75	10.000	0.900
	< 2.715	100	54.07 - 100	80	44.39 - 97.48	5.000	0.800
	< 3.055	100	54.07 - 100	70	34.75 - 93.33	3.333	0.700
	< 3.135	100	54.07 - 100	60	26.24 - 87.84	2.500	0.600
	< 3.295	100	54.07 - 100	50	18.71 - 81.29	2.000	0.500
	< 3.455	100	54.07 - 100	40	12.16 - 73.76	1.667	0.400

	< 3.655	100	54.07 - 100	30	6.67 - 65.25	1.429	0.300
	< 3.905	100	54.07 - 100	20	2.52 - 55.61	1.250	0.200
	< 4.035	100	54.07 - 100	10	0.25 - 44.50	1.111	0.100
miR-125b-5p	< 1.205	16.67	0.42 - 64.12	100	69.15 - 100		0.167
	< 1.980	33.33	4.33 - 77.72	100	69.15 - 100		0.333
	< 2.760	50	11.81 - 88.19	100	69.15 - 100		0.500
	< 3.030	50	11.81 - 88.19	90	55.50 - 99.75	5.000	0.400
	< 3.280	83.33	35.88 - 99.58	90	55.50 - 99.75	8.333	0.733
	< 3.810	100	54.07 - 100	90	55.50 - 99.75	10.000	0.900
	< 4.265	100	54.07 - 100	80	44.39 - 97.48	5.000	0.800
	< 4.540	100	54.07 - 100	70	34.75 - 93.33	3.333	0.700
	< 4.725	100	54.07 - 100	60	26.24 - 87.84	2.500	0.600
	< 4.835	100	54.07 - 100	50	18.71 - 81.29	2.000	0.500
	< 4.935	100	54.07 - 100	40	12.16 - 73.76	1.667	0.400
	< 5.015	100	54.07 - 100	30	6.67 - 65.25	1.429	0.300
	< 5.170	100	54.07 - 100	20	2.52 - 55.61	1.250	0.200
	< 5.340	100	54.07 - 100	10	0.25 - 44.50	1.111	0.100
miR-192-5p	> 0.520	0	0 - 30.85	83.33	35.88 - 99.58	0.833	-0.167
	> 1.470	0	0 - 30.85	66.67	22.28 - 95.67	0.667	-0.333
	> 2.405	10	0.25 - 44.50	66.67	22.28 - 95.67	0.741	-0.233
	> 2.630	20	2.52 - 55.61	66.67	22.28 - 95.67	0.833	-0.133
	> 2.795	30	6.67 - 65.25	66.67	22.28 - 95.67	0.952	-0.033
	> 2.955	40	12.16 - 73.76	66.67	22.28 - 95.67	1.111	0.067
	> 3.210	40	12.16 - 73.76	50	11.81 - 88.19	0.833	-0.100
	> 3.435	50	18.71 - 81.29	50	11.81 - 88.19	1.000	0.000
	> 3.605	60	26.24 - 87.84	50	11.81 - 88.19	1.250	0.100
	> 3.730	70	34.75 - 93.33	50	11.81 - 88.19	1.667	0.200
	> 3.865	80	44.39 - 97.48	50	11.81 - 88.19	2.500	0.300
	> 4.040	90	55.50 - 99.75	50	11.81 - 88.19	5.000	0.400
	> 4.180	90	55.50 - 99.75	33.33	4.33 - 77.72	3.333	0.233
	> 4.280	90	55.50 - 99.75	16.67	0.42 - 64.12	1.667	0.067
	> 4.450	90	55.50 - 99.75	0	0 - 45.93	0.000	-0.100

AHEv = viremic acute; CI = confidence interval

Supplementary Table S3 Optimal cut-off values for miR-99a-5p, miR-125b-5p and miR-192-5p in serum samples from CHEv patients when compared to non-infected control patients as determined by Youden's Index for ROC analysis

			CHEv				
miRNA	Cut-off	Sensitivity [%]	95% CI [%]	Specificity [%]	95% CI [%]	Likelihood Ratio	Youden's J
miR-99a-5p	< 0.995	8.33	0.21 - 38.48	100	69.15 - 100		0.083
	< 1.190	16.67	2.09 - 48.41	100	69.15 - 100		0.167
	< 1.380	25	5.49 - 57.19	100	69.15 - 100		0.250
	< 1.415	33.33	9.93 - 65.11	100	69.15 - 100		0.333

	< 1.440	33.33	9.93 - 65.11	90	55.50 - 99.75	3.333	0.233
	< 1.585	41.67	15.17 - 72.33	90	55.50 - 99.75	4.167	0.317
	< 1.765	50	21.09 - 78.91	90	55.50 - 99.75	5.000	0.400
	< 1.875	58.33	27.67 - 84.83	90	55.50 - 99.75	5.833	0.483
	< 2.025	66.67	34.89 - 90.08	90	55.50 - 99.75	6.667	0.567
	< 2.115	75	42.81 - 94.51	90	55.50 - 99.75	7.500	0.650
	< 2.280	83.33	51.59 - 97.91	90	55.50 - 99.75	8.333	0.733
	< 2.500	83.33	51.59 - 97.91	80	44.39 - 97.48	4.167	0.633
	< 2.650	91.67	61.52 - 99.79	80	44.39 - 97.48	4.583	0.717
	< 2.865	100	73.54 - 100	80	44.39 - 97.48	5.000	0.800
	< 3.055	100	73.54 - 100	70	34.75 - 93.33	3.333	0.700
	< 3.135	100	73.54 - 100	60	26.24 - 87.84	2.500	0.600
	< 3.295	100	73.54 - 100	50	18.71 - 81.29	2.000	0.500
	< 3.455	100	73.54 - 100	40	12.16 - 73.76	1.667	0.400
	< 3.655	100	73.54 - 100	30	6.67 - 65.25	1.429	0.300
	< 3.905	100	73.54 - 100	20	2.52 - 55.61	1.250	0.200
	< 4.035	100	73.54 - 100	10	0.25 - 44.50	1.111	0.100
miR-125b-5p	< 2.420	8.33	0.21 - 38.48	100	69.15 - 100		0.083
	< 2.675	16.67	2.07 - 48.41	100	69.15 - 100		0.167
	< 2.920	25	5.49 - 57.19	100	69.15 - 100		0.250
	< 3.060	25	5.49 - 57.19	90	55.50 - 99.75	2.500	0.150
	< 3.165	33.33	9.93 - 65.11	90	55.50 - 99.75	3.333	0.233
	< 3.245	41.67	15.17 - 72.33	90	55.50 - 99.75	4.167	0.317
	< 3.480	50	21.09 - 78.91	90	55.50 - 99.75	5.000	0.400
	< 3.775	58.33	27.67 - 84.83	90	55.50 - 99.75	5.833	0.483
	< 3.935	66.67	34.89 - 90.08	90	55.50 - 99.75	6.667	0.567
	< 4.005	75	42.81 - 94.51	90	55.50 - 99.75	7.500	0.650
	< 4.065	83.33	51.59 - 97.91	90	55.50 - 99.75	8.333	0.733
	< 4.255	83.33	51.59 - 97.91	80	44.39 - 97.48	4.167	0.633
	< 4.400	91.67	61.52 - 99.79	80	44.39 - 97.48	4.583	0.717
	< 4.415	91.67	61.52 - 99.79	70	34.75 - 93.33	3.056	0.617
	< 4.545	100	73.54 - 100	70	34.75 - 93.33	3.333	0.700
	< 4.725	100	73.54 - 100	60	26.24 - 87.84	2.500	0.600
	< 4.835	100	73.54 - 100	50	18.71 - 81.29	2.000	0.500
	< 4.935	100	73.54 - 100	40	12.16 - 73.76	1.667	0.400
	< 5.015	100	73.54 - 100	30	6.67 - 65.25	1.429	0.300
	< 5.170	100	73.54 - 100	20	2.52 - 55.61	1.250	0.200
	< 5.340	100	73.54 - 100	10	0.25 - 44.50	1.111	0.100
miR-192-5p	< 1.235	8.33	0.21 - 38.48	100	69.15 - 100		0.083
	< 1.385	25	5.47 - 57.19	100	69.15 - 100		0.250
	< 1.645	33.33	9.93 - 65.11	100	69.15 - 100		0.333
	< 1.980	41.67	15.17 - 72.33	100	69.15 - 100		0.417
	< 2.100	58.33	27.67 - 84.83	100	69.15 - 100		0.583
	< 2.230	66.67	34.89 - 90.08	100	69.15 - 100		0.667
	< 2.380	66.67	34.89 - 90.08	90	55.50 - 99.75	6.667	0.567
	< 2.445	75	42.81 - 94.51	90	55.50 - 99.75	7.500	0.650
	< 2.510	75	42.81 - 94.51	80	44.39 - 97.48	3.750	0.550
	< 2.555	91.67	61.52 - 99.79	80	44.39 - 97.48	4.583	0.717
	< 2.675	100	73.54 - 100	80	44.39 - 97.48	5.000	0.800
	< 2.795	100	73.54 - 100	70	34.75 - 93.33	3.333	0.700

	< 3.055	100	73.54 - 100	60	26.24 - 87.84	2.500	0.600
	< 3.435	100	73.54 - 100	50	18.71 - 81.29	2.000	0.500
	< 3.605	100	73.54 - 100	40	12.16 - 73.76	1.667	0.400
	< 3.730	100	73.54 - 100	30	6.67 - 65.25	1.429	0.300
	< 3.865	100	73.54 - 100	20	2.52 - 55.61	1.250	0.200
	< 4.230	100	73.54 - 100	10	0.25 - 44.50	1.111	0.100

CHEv = viremic chronic; CI = confidence interval

Supplementary Fig. S4 ROC curves for miR-99a-5p, miR-125-5p and miR-192-5p in AHEv (A-C) and CHEv (D-F) patients compared to non-infected control patients were calculated. Dotted lines indicate fixed values of 90% specificity and 90% sensitivity, respectively, of the model. Chance level is represented by a dashed line. ROC AUC values are presented in Table 6

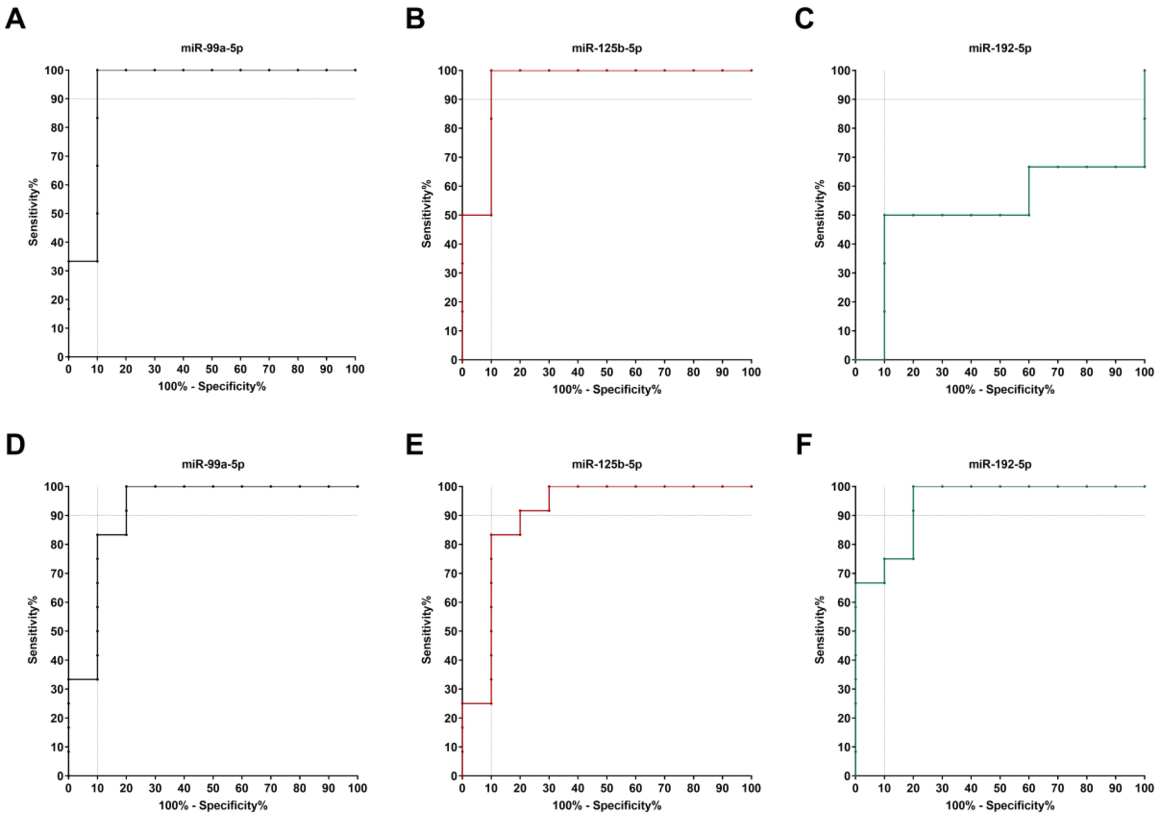

Supplementary Fig. S5 Correlation analysis of serum miR-122-5p expression and ALT (A) and AST (B) levels in CHE and non-infected control patients. Each dot represents on patient

sample (blue = CHEv; red = CHEnv; green = control). miR-122-5p ΔCt values are plotted on x-axis while ALT and AST levels, respectively, are plotted on y-axis. Pearson correlation coefficient was calculated and plotted for each model. Correlation analysis values are presented in Table 7

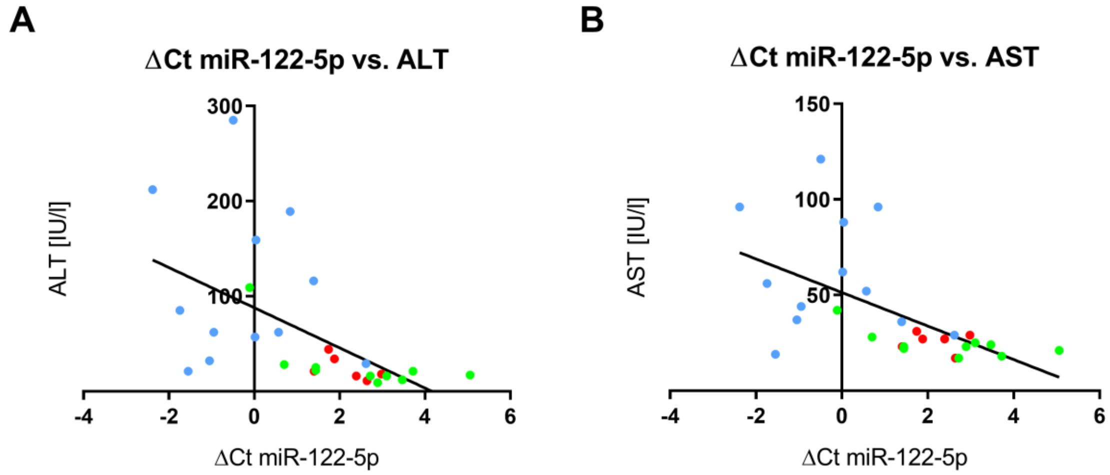

Supplement: Supplementary file 1 — Dataset 1. [file 41598_2020_62159_MOESM1_ESM.pdf]
